# IL-22 Is Mainly Produced by IFNγ-Secreting Cells but Is Dispensable for Host Protection against *Mycobacterium tuberculosis* Infection

**DOI:** 10.1371/journal.pone.0057379

**Published:** 2013-02-27

**Authors:** Jochen Behrends, Jean-Christophe Renauld, Stefan Ehlers, Christoph Hölscher

**Affiliations:** 1 Infection Immunology, Research Center Borstel (RCB), Borstel, Germany; 2 Ludwig Institute for Cancer Research and Experimental Medicine Unit, Université Catholique de Louvain, Brussels, Belgium; 3 Microbial Inflammation Research, RCB, Borstel, Germany; 4 Molecular Inflammation Medicine, Christian-Albrechts-University, Kiel, Germany; 5 Cluster of Excellence Inflammation-at-Interfaces (Borstel-Kiel-Lübeck-Plön), Germany; University of Cape Town, South Africa

## Abstract

Anti-inflammatory treatment of autoimmune diseases is associated with an increased risk of reactivation tuberculosis (TB). Besides interleukin (IL-17)A, IL-22 represents a classical T helper (TH)17 cytokine and shares similar pathological effects in inflammatory diseases such as psoriasis or arthritis. Whereas IL-17A supports protective immune responses during mycobacterial infections, the role of IL-22 after infection with *Mycobacterium tuberculosis* (*Mtb*) is yet poorly characterized. Therefore, we here characterize the cell types producing IL-22 and the protective function of this cytokine during experimental TB in mice. Like IL-17A, IL-22 is expressed early after infection with *Mtb* in an IL-23-dependent manner. Surprisingly, the majority of IL-22-producing cells are not positive for IL-17A but have rather functional characteristics of interferon-gamma-producing TH1 cells. Although we found minor differences in the number of naive and central memory T cells as well as in the frequency of TH1 and polyfunctional T cells in mice deficient for IL-22, the absence of IL-22 does not affect the outcome of *Mtb* infection. Our study revealed that although produced by TH1 cells, IL-22 is dispensable for protective immune responses during TB. Therefore, targeting of IL-22 in inflammatory disease may represent a therapeutic approach that does not incur the danger of reactivation TB.

## Introduction

Reactivation of latent tuberculosis (TB) after therapeutic tumor necrosis factor (TNF) blockade during chronic inflammatory diseases such as rheumatoid arthritis highlights TNF as an essential mediator containment of infection with *Mycobacterium tuberculosis* (*Mtb*) [1]–[4]. Therefore, current inflammation research aims at discovering new targets that selectively block disease-promoting mechanisms during inflammatory diseases without compromising protective immune responses against *Mtb*.

Innate immune cells alone are not capable to control *Mtb* infection [5] but the induction of an acquired immune response is essential for a protective host immune defense of *Mtb* [6], [7]. Particularly the interleukin (IL)-12-mediated differentiation of T helper (TH)1 cells promote the production of interferon-gamma (IFNγ) and TNF, which in turn are essential to activate effector mechanisms in macrophages for controlling the intracellular growth of *Mtb* [8]–[10]. In addition, these cytokines also play an important role in the formation of granulomas, a well-structured accumulation of immune cells required for the containment of mycobacterial replication [11]–[13], that may also contribute to immunopathology and bacterial dissemination [14]–[16].

In contrast to IFNγ- and TNF-secreting TH1 cells, TH17 cells are defined by their production of IL-17A, IL-17F, IL-21, and IL-22 [17], [18]. After infection with *Mtb*, the proliferation of IL-17A-producing cells is dependent on IL-23, which mediates protective mechanisms in the absence of IL-12 [19]–[22]. IL-17A contributes to the host’s immune defense by the induction of chemokines and cytokines that are responsible for the early recruitment of neutrophils and granuloma formation [23], [24]. Furthermore, IL-17A is involved in the establishment of protective TH1 cell responses after vaccination against *Mtb* [25], [26]. In contrast to this protective function, overproduction of IL-17A may also have pathological effects during *Mtb*-induced inflammation [27]. After *Mtb* infection, IL-17A is mainly produced by γδT cells rather than CD4^+^ T cells [28]. The differential role of these two T cell populations still remains elusive [29].

The imponderability of different cytokines towards protection or pathology during inflammation is even greater if another TH17 cytokine, IL-22 is considered. IL-22 is a member of the IL-10 family, mainly produced by T cells and natural killer (NK) cells and represents an effector cytokine of the TH17 lineage [30]–[33] that mediates immunopathology in inflammatory diseases, such as psoriasis or arthritis [34]–[36]. IL-22 acts through a receptor complex consisting of the private IL-22 receptor type I and the IL-10 receptor (R)2 subunits that is expressed on various cell types such as keratinocytes and fibroblasts. It induces pro-inflammatory chemokines and cytokines, antimicrobial peptides and proteins involved in tissue remodeling [33], [37]–[40]. However, IL-22 shares some downstream effects with IL-10 [38], [41] and can act immunosuppressive during airway inflammation through an IL-10 associated mechanism [42]. Overall, targeting IL-22 or its receptors represents a promising approach to ameliorate the outcome of autoimmune diseases such as psoriasis or psoriatic [43], [44]. However, the treatment of such diseases with anti-inflammatory drugs has been associated with reactivation of latent TB [45], [46]. To evaluate the potential risk of interfering with IL-22-dependent inflammation on the outcome of *Mtb* infection, we analyzed IL-22-deficient (^−/−^) mice in experimental pulmonary TB, because the impact of IL-22 on protective immune responses during mycobacterial infections is currently poorly understood. In human macrophages NK cell-derived IL-22 inhibits the intracellular growth of *Mtb* [47], [48]. In a murine aerosol model, IL-22 has been shown to contribute to chemokine (C-X-C motif) ligand (CXCL)13-mediated B cell follicle organization in granulomas, which appears to have a protective function during *Mtb* infection [48]–[52]. However, neutralization of IL-22 has no influence on the outcome of experimental TB [53]. Together, after infection with *Mtb* the cellular sources of this TH17 cytokine are not defined and the impact of IL-22 on protective immune responses is not clearly understood yet. To evaluate whether therapeutic targeting of IL-22 represents a promising approach for the therapy of autoimmune diseases without compromising cell-mediated immunity in *Mtb* infection, we here determined the IL-22-producing cell types and determined the functional and protective significance of IL-22 using IL-22^−/−^ mice in an experimental aerosol model of TB.

## Materials and Methods

### Mice

IL-22^−/−^ mice were bred and maintained under specific-pathogen-free conditions at the Research Center Borstel. IL-23p19^−/−^ mice were obtained from the Institute of Animal Breeding and Husbandry at the Christian-Albrechts-University (Kiel, Germany) and C57BL/6 mice (Charles River, Sülzfeld, Germany) were used as controls. Experimental mice were between 8 and 16 weeks old. In any given experiment, mice were matched for age, sex and genetic background. For infection experiments, mice were kept under barrier conditions in the BSL 3 facility at the Research Center Borstel (Borstel, Germany) in individually ventilated cages. All experiments performed were in accordance with the German Animal Protection Law and were approved by the Animal Research Ethics Board of the Ministry of Environment, Kiel, Germany.

### Bacteria and Infection

For infection experiments, *Mtb* H37Rv were used. *Mtb* was grown in Middlebrook 7H9 broth (Difco, Detroit, MI) supplemented with Middlebrook OADC enrichment medium (Life Technologies, Gaithersburg, MI), 0.002% glycerol, and 0.05% Tween 80. Midlog phase cultures were harvested, aliquoted, and frozen at -80°C. After thawing, viable cell counts were determined by plating serial dilutions of the cultures on Middlebrook 7H10 agar plates followed by incubation at 37°C. Before infection of experimental animals, stock solutions of *Mtb* were diluted in sterile distilled water and pulmonary infection was performed using an inhalation exposure system (Glas-Col, Terre-Haute, IN). To infect mice with a low dose (100–200 CFU/lung) or high dose (1000–2000 CFU/lung), animals were exposed for 40 min to an aerosol generated by nebulising approximately 5.5 ml of a suspension containing 10^5^–10^7^ live bacteria. Inoculum size was checked 24 h after infection by determining the bacterial load in the lung of infected mice.

### Colony Enumeration Assay

Bacterial loads in lungs, spleen and liver were evaluated at different time points after infection with *Mtb* to follow the course of infection. Organs from sacrificed animals were removed aseptically, weighed and homogenized in PBS containing a proteinase inhibitor cocktail (Roche Diagnostics, Mannheim, Germany) prepared according to the manufacturer’s instructions. Tenfold serial dilutions of organ homogenates were plated in duplicates onto Middlebrook 7H10 agar plates containing 10% OADC and incubated at 37°C for 21–25 days. Colonies on plates were enumerated and results expressed as log_10_ CFU per organ.

### Preparation of Single Cell Suspensions from Infected Lungs

For antigen-specific restimulation and flow cytometric analysis, single cell suspensions of lungs were prepared from *Mtb*-infected mice at different time points. Mice were anesthetized and injected intraperitoneally with 150 U Heparin (Ratiopharm, Ulm, Germany). Lungs were perfused through the right ventricle with warm PBS. Once lungs appeared white, they were removed and sectioned. Dissected lung tissue was then incubated in collagenase A (0.7 mg/ml; Roche Diagnostics, Mannheim, Germany) and DNase (30 µg/ml; Sigma) at 37°C for 2 h. Digested lung tissue was gently disrupted by subsequent passage through a 100 µm pore size nylon cell strainer. Recovered vital lung cells were counted using an automatic cell counter (ViCell®; Beckman Coulter, Krefeld, Germany), diluted in complete Iscove’s-modified Dulbecco’s medium (IMDM; Gibco) supplemented with 10% FCS (Gibco), 0.05 mM β-mercaptoethanol (Sigma, Deisenhofen, Germany), and penicillin and streptomycin (100 U/ml and 100 µg/ml; Gibco) and used for further experiments.

### Flow Cytometric Analysis

For flow cytometric analysis of surface markers and intracellular cytokines, enriched CD4^+^ T cells, and single-cell suspensions of lungs were stained with optimal concentrations of the following specific antibodies against surface molecules and intracellular cytokines: CD8 α-V450, CD4-V500, CD11c-FITC, CD62L-APC, Gr-1-APC, IFNγ-APC from BD Biosciences, CD11b-PerCP-Cy5.5, IL-17A-PerCP-Cy5.5, CD90.2-PE-Cy7, CD90.2-eFluor780, and MHCII(I-A/I-E)-eFluor780 from eBioscience, CD44-FITC, and IFNγ-PE from BioLegend, and IL-22-PE from R&D Systems. Fluorescence intensity was measured by using a FacsCantoII® flow cytometer (BD Bioscience) equipped with a 405 nm, 488 nm and 633 nm laser. Analysis was performed utilizing the FCSExpress4 program (DeNovo™ Software) gated on leukocytes identified by the forward-scatter/side-scatter profile and further characterization as specified in the figures.

### RT-PCR

Weighed lung samples before and at different time points after aerosol infection with *Mtb*, were homogenized in 4 M guanidinium-isothiocyanate buffer and total RNA was extracted by acid phenol extraction. cDNA was obtained using murine moloney leukemia virus (MMLV) reverse transcriptase (Superscript II, Invitrogen, Karlsruhe, Germany) and oligo-dT (12–18mer; Sigma) as a primer. Quantitative PCR was performed on a Light Cycler (Roche Diagnostics Corporation, Indianapolis, IN). Data were analyzed employing the “Fit Points” and “Standard Curve Method” using hypoxanthine-guanine phosphoribosyle transferase (*Hprt*) as housekeeping gene to calculate the level of gene expression in relation to *Hprt*. The following primer and probe sets were employed:

*Hprt*: sense 5′-TCC TCC TCA GAC CGC TTT T-3′, antisense 5′-CCT GGT TCA TCA TCG CTA ATC-3′, probe 5′-AGT CCA G-3′; *Il-22*: sense 5′-TTT CCT GAC CAA ACT CAG CA-3′, antisense 5′ TCT GGA TGT TCT GGT CGT CA-3′, probe 5′-CAG CTC CT-3′; *Il-6*: sense 5′-GCT ACC AAA CTG GAT ATA ATC AGG A-3′, antisense 5`CCA GGT AGC TAT GGT ACT CCA GAA-3′, probe 5′-TTC CTC TG-3′; *Il-12b*: sense 5′-ATC GTT TTG CTG GTG TCT CC -3′, antisense 5′-GGA GTC CAG TCC ACC TCT ACA-3′, probe 5′-AGC TGG AG-3′; *Ifng*: sense 5′-ATC TGG AGG AAC TGG CAA AA-3′, antisense 5′-TTC AAG ACT TCA AAG AGT CTG AGG TA-3′, probe 5′-CAG AGC CA-3′; *Nos2*: sense 5′-CTT TGC CAC GGA CGA GAC-3′, antisense 5′-GTC ATT GTA CTC TGA GGG CTG A-3′, probe 5′-AGG CAG AG-3′; *Lrg47*: sense 5′-AAG GCC ACT AAC ATC GAA TCA-3′, antisense 5′-TGC CTT ATC TCA CTT AAT ACT CCT CA-3′, probe 5′-CTC CTC TG-3′; *Il-17a*: sense 5′-TGT GAA GGT CAA CCT CAA AGT CT-3′, antisense 5′-GAG GGA TAT CTA TCA GGG TCT TCA T-3′, probe 5′-GCT CCA GA-3′; *Il-17f*: sense 5′-CCC AGG AAG ACA TAC TTA GAA GAA A-3′, antisense 5′-CAA CAG TAG CAA AGA CTT GAC CA-3′, probe 5′-ATG GCT GC-3′; *Tnf*: sense 5′-TCT TCT CAT TCC TGC TTG TGG-3′, antisense 5′-GGT CTG GGC CAT AGA ACT GA-3′, probe 5′-TGG TGG CC-3′; *Il-10*: sense 5′-CAG AGC CAC ATG CTC CTA GA-3′, antisense 5′-GTC CAG CTG GTC CTT TGT TT-3′, probe 5′-CTT CAG CC-3′. Additionally PCR was performed using the proprietary Light-Cycler-DNA Master SYBR Green® I kit (Roche Diagnostics) and the following primer sets:

*Hprt*: sense 5′-GCA GTA CAG CCC CAA AAT-3′, antisense 5′-AAC AAA GTC TGG CCT GTA TCC AA-3′; *Il-23p19*: sense 5′-TCC CTA CTA GGA CTC AGC CAA C-3′, antisense 5′-TGG GCA TCT GTT GGG TCT-3′. After amplification, melting curve analysis was performed to exclude the presence of confounding primer-dimers during quantification. For purposes of statistical comparison, units were normalized by calculating the cDNA ratios of gene-of-interest/*Hprt* for each sample.

### Intracellular Cytokine Staining

For detection of intracellular IFNγ, IL-22 and IL17A an intracellular cytokine staining kit was employed (BD Biosciences). Briefly, single cell suspensions were prepared at 22–254 days after infection and 1.8×10^6^ cells were incubated without or with plate-bound anti-CD3/CD28 mAb (clone 2C11 and clone 37/51 at 5 µg/ml, respectively) for 4.5 h in the presence of GolgiPlug™ (BD Biosciences). Non-specific antibody binding was blocked by incubation with a cocktail containing anti-FcγRIII/II mAb (clone 2.4G2), mouse, hamster and rat serum. Cells were washed and incubated with optimal concentrations of V450, V500, FITC and PE-Cy7 labelled mAbs directed against CD8α and CD4 (BD Biosciences), CD44 (BioLegend), and CD90.2 (eBioscience). After staining, cells were fixed and permeabilized with Cytofix/Cytoperm™ (BD Biosciences) and intracellularly accumulated IL-22, IL-17A, and IFNγ were stained with PE (R&D Systems), PerCP-Cy5.5 (eBioscience), and APC (BD Biosciences) labelled mAbs. IgG2a-PE, IgG2a-PerCP-Cy5.5 and IgG1-APC antibodies were applied for isotype control stainings. Fluorescence intensity was analysed on a FacsCantoII® (BD Biosciences) gated as described in the figures.

### ESAT6_1-20_-specific ELISPOT Assays

For measuring antigen-specific CD4^+^ T cells in lungs from infected mice, single cell suspension were resuspended in IMDM. To enrich CD4^+^ T cells, single cell suspensions were incubated with magnetic CD4 microbeads (Miltenyi, Bergisch Gladbach, Germany) and separated from other cells on a MACS separation unit (Miltenyi). Separated CD4^+^ T cells were collected in IMDM, counted using a cell counter (ViCell®; Beckman Coulter), diluted in IMDM and used for further experiments. Purity of enriched CD4^+^ T cells was >90% as determined by flow cytometry. Detection of antigen-specific IFNγ- and IL-17-A producing CD4^+^ T cells from infected lungs was conducted using ELISPOT assay kits (BD Bioscience and R&D Systems, Wiesbaden-Nordenstadt, Germany, respectively). In brief, purified CD4^+^ T cells or single cell suspensions from lungs of infected mice were seeded in wells of anti-mouse IFNγ- or IL-17-A coated and blocked 96-well multi test plates at an initial concentration of 1×10^5^ cells/well in IMDM. After doubling dilutions of these cells were made, mitomycin-D (Sigma)-inactivated splenocytes from uninfected wild-type mice were used as APCs at a concentration of 1×10^6^ cells/well. CD4^+^ T cells and single cell suspensions were restimulated with the *Mtb* ESAT6_1–20_ (Research Center Borstel, Germany) at a concentration of 10 µg/ml in the presence of 10 U/ml recombinant mouse IL-2 (Peprotech). After 20 h of incubation in 5% CO_2_ at 37°C, plates were washed, and biotinylated anti-mouse IFNγ was used to detect the captured cytokine. Spots were visualized using streptavidin-HRP as substrate. After 24 h of incubation in 5% CO_2_ at 37°C, plates were washed, and biotinylated anti-mouse IL-17A was used to detect the captured cytokine. Spots were visualized using streptavidin-AP as substrate. Spots were automatically enumerated using an ELISPOT reader (EliSpot 04 XL; AID). The frequency of responding CD4^+^ T cells or responding cells from the single cell suspensions was determined. Neither CD4^+^ T cells or single cell suspension cells cultured in the absence of ESAT6_1–20_ nor cells from uninfected mice produced detectable spots.

### Statistical Analysis

Quantifiable data are expressed as means of individual determinations and standard errors. Statistical analysis was used with the Prism software program (GraphPad Software) to perform two-tailed Student *t* test or two-way ANOVA and Bonferroni’s multiple comparison post-test as appropriate. Differences were considered significant when p<0.05. The log-rank (Mantel-Cox) test was used for statistical analysis for survival experiments.

## Results

### After *Mtb* Infection, IL-22 Expression is Dependent on IL-23

**Figure 1:**
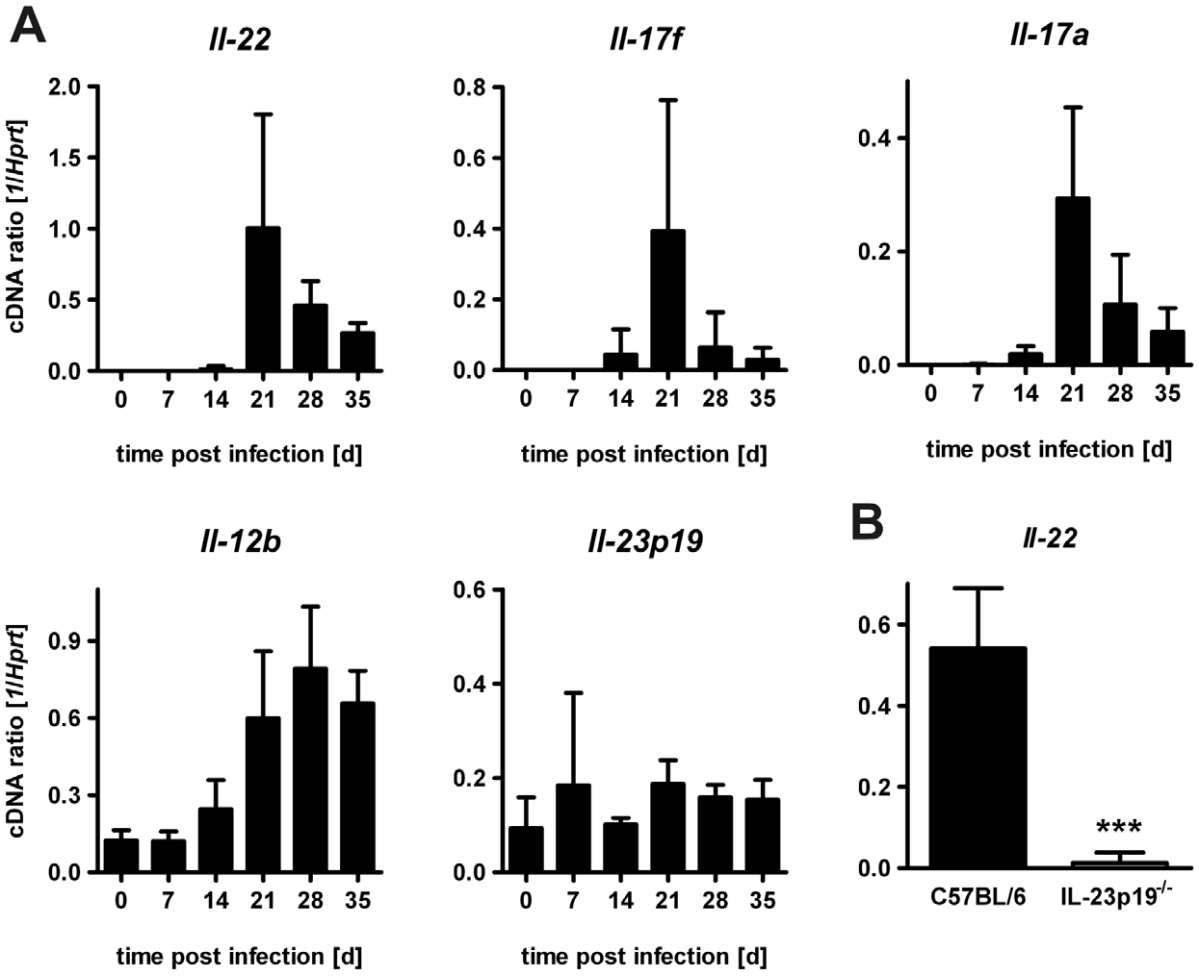
After *Mtb* infection, IL-22 expression is IL-23 dependent. Experimental mice were infected with *Mtb* via the aerosol route. (**A**) Before and at different time points of infection with approx. 100 CFU *Mtb*, gene expression of *Il22*, *Il12b*, *Il17a*, *Il17f*, and *Il23p19* was quantified by real time RT-PCR in lung homogenates of C57BL/6 mice based on the expression of *Hprt*. Data represent mean ± SD of 5 mice per group. (**B**) Twenty days after infection with approx. 1000 CFU *Mtb* of C57BL/6 (black symbols) and IL-23p19^−/−^ (white symbols) mice, gene expression of *Il22* was quantified by real time RT-PCR in lung homogenates based on the expression of *Hprt*. Data represent mean ± SD for of 5 mice per group. Statistical analysis was performed using the Student’s *t* test defining differences between C57BL/6 and IL-23p19^−/−^ mice as significant (***, p<0.001).

Early after infection of C57BL/6 mice with *Mtb* real time RT-PCR of lung homogenates was conducted to compare gene expression of *Il22* with the expression of *Il17a*, *Il17f*, *Il12b*, and *Il23p19* (Figure 1A). Uninfected mice expressed very low mRNA levels of these cytokines. Gene expression of *Il22, Il17a*, *Il17f*, and *Il12b* started to increase as early as day 14 of *Mtb* infection. Maximal expression of *Il22, Il17a*, and *Il17f* were found at day 21 after which mRNA levels decreased to negligible amounts at day 35. In contrast, *Il12b* gene expression increased starting at day 14 with a plateau 28 days after *Mtb* infection. Gene expression of *Il23p19* was not induced after an infection with *Mtb*. IL-22 has been shown to be co-expressed in TH17 cells with IL-17A [32]. Because after *Mtb* infection IL-17A production is mediated by IL-23 [20], we determined whether *Mtb*-infected IL-23p19^−/−^ were also deficient in IL-22 expression. Quantitative real time RT-PCR of lung homogenates from C57BL/6 and IL23p19^−/−^ mice infected with *Mtb* for 21 days revealed that *Il22* gene expression is promoted by IL-23 (Figure 1B). These results show that after infection with *Mtb* IL-22 is expressed with very similar kinetics and in a comparable IL-23-dependent manner to other TH17 cytokines such as IL-17A and IL-17F.

### During Experimental TB, IL-22 is Mainly Expressed by IFNγ-producing Cells

**Figure 2:**
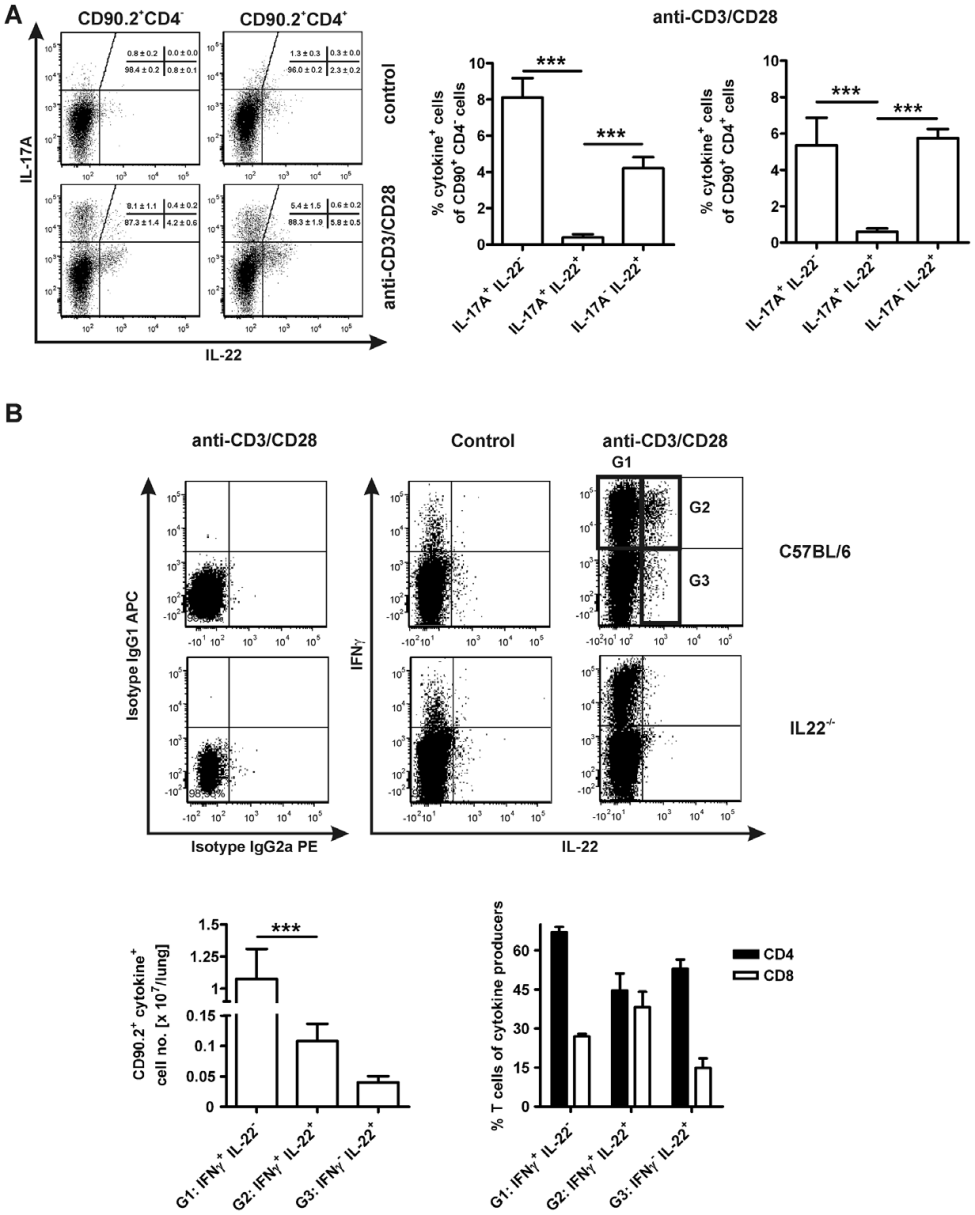
In experimental TB, IL-22 is mainly expressed by IFNγ-producing cells. Experimental mice were infected with approx. 100 CFU *Mtb* via the aerosol route. (**A**, **B**). After 22 days of infection, lung cells of C57BL/6 mice were restimulated with anti-CD3/CD28 or left unstimulated. Cells were subsequently stained for CD90.2, CD4, IL-17A, IFNγ and IL-22, analyzed by flow cytometry and the frequencies of cytokine-producing cells were compared. (**A**) Representative dot plots and frequencies of cytokine-producing CD4^neg^ (left panel, left graph) and CD4^+^ (right panel, right graph) cells gated for CD90.2^+^. The frequencies of gated cytokine-producing cells are shown and represent means ± SD of 5 mice. (**B**) Representative dot plots of control stainings (*i*, isotype controls; *ii*, unstimulated cells; *iii*, staining in IL-22^−/−^ mice) and of cytokine-producing cells indicating G1 (IFNγ^+^IL-22^neg^), G2 (IFNγ^+^IL-22^+^) and G3 (IFNγ^neg^IL-22^+^) for further analysis. The absolute numbers and frequencies of gated cytokine-producing cells are shown and represent means ± SD of 5 mice. Statistical analysis was performed using two-way ANOVA defining differences between different cytokine-producing cell populations as significant (***, p<0.001).

It is assumed that IL-22 is co-expressed in TH17 cells with IL-17A [32]. To directly assess the question which cell types produce IL-22 during experimental TB, C57BL/6 mice were infected with a low dose of *Mtb* for 22 days, and the production of IL-17A, IFNγ and IL-22 in CD90.2^+^CD4^+^ cells was analysed by flow cytometry after restimulation of lung cells with anti-CD3/CD28 and intracellular cytokine staining (Figure 2). The analysis encompasses four distinct CD90.2^+^ cytokine-producing populations (Figure 2A): IL-17A^+^ or IL-22^+^ cells negative for CD4, and CD4^+^ cells, which were capable to produce IL-17A^+^ or IL-22^+^. The frequency of IL-17A producing cells was enhanced in the CD90.2^+^CD4^neg^ population (8.1%) when compared to CD90.2^+^CD4^+^ cells (5.4%), but the relative amount of IL-22-producing cells were higher in the CD90.2^+^CD4^+^ population (5.8%) compared to CD90.2^+^CD4^neg^ cells (4.2%). Interestingly, after restimulation, the frequency of IL-17A-producing cells was approx. 10-fold higher in CD90.2^+^CD4^neg^ cells compared to an only 5-fold increased relative amount of IL-17A^+^CD90.2^+^CD4^+^ cells. Moreover, the frequency of IL-22^+^CD90.2^+^CD4^neg^ cells increased to a higher extent (approx. 5-fold) when compared to IL-22^+^CD90.2^+^CD4^+^ cells (approx. 3-fold). CD90.2^+^CD4^+^ cells that only produce IL-22 were hardly detectable in lungs of *Mtb*-infected mice (0.6%). Surprisingly, most IL-22-producing cells were found not to be positive for IL-17A (approx. 90%).

Therefore, we further characterised cells producing IL-22 during experimental TB and surprisingly found that the majority of IL-22^+^CD90.2^+^ cells simultaneously expressed IFNγ (Figure 2B). IFNγ^+^IL-22^neg^ (G1) cells were inducible upon stimulation (approx. 14-fold) and consisted of 67% CD4^+^ and 27% CD8^+^ cells. IFNγ^+^IL-22^+^ (G2) cells showed a very low intracellular accumulation of cytokines when left unstimulated and cytokine expression was strongly enhanced after restimulation (approx. 59-fold). CD4^+^ cells represent 45% of this population and 38% were found positive for CD8. In contrast to these IFNγ-expressing populations, IFNγ^neg^IL-22^+^ (G3) cells could hardly be restimulated with anti-CD3/CD28 (approx. 1.4-fold) and consisted of 53% CD4^+^ and 15% CD8^+^ cells. Together, we found that during experimental TB IL-22 is mainly produced by IFNγ-expressing CD4^+^ T cells.

### The Inflammatory Cytokine Responses in IL-22^−/−^ Mice after Low Dose *Mtb* Infection

**Figure 3:**
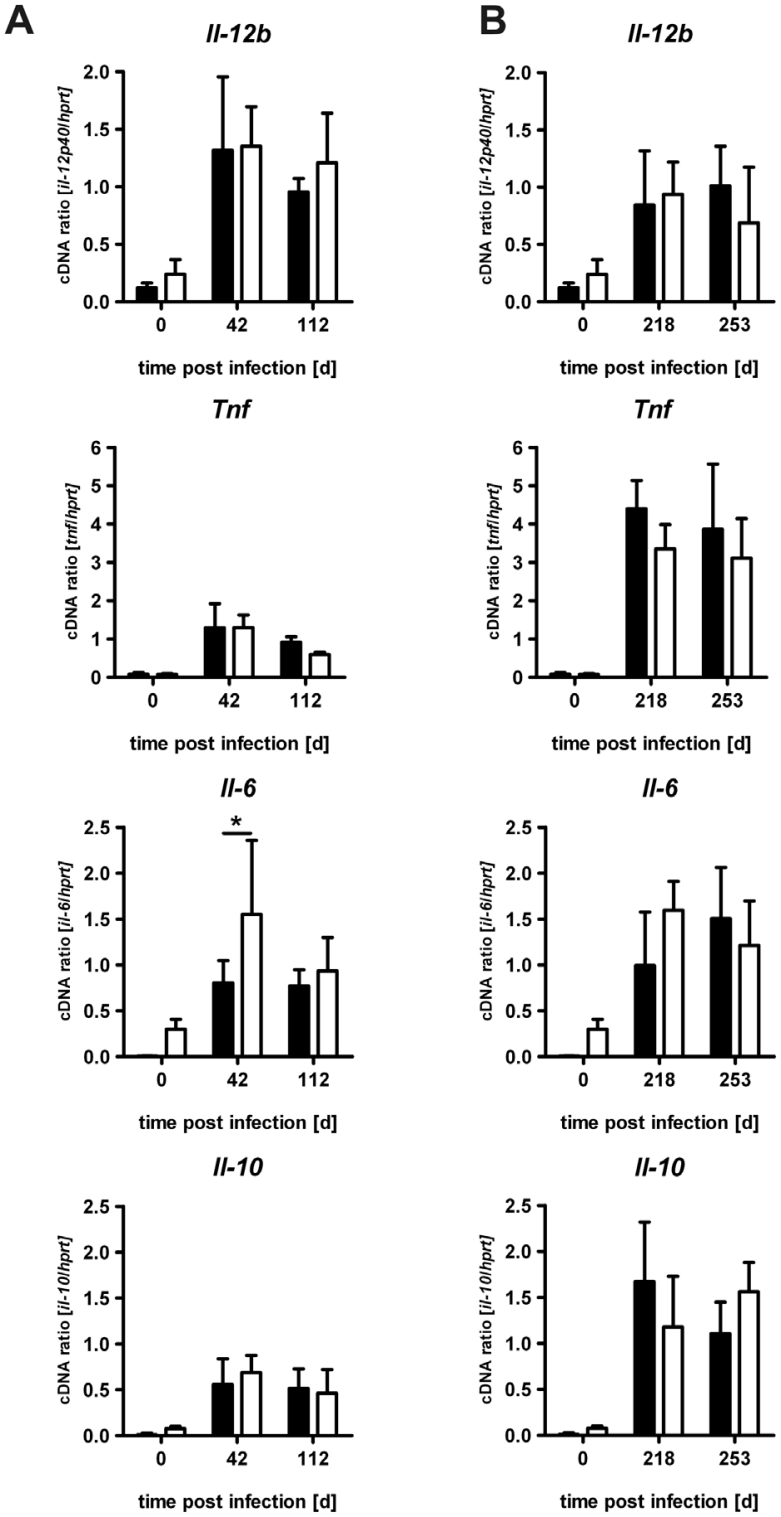
The inflammatory cytokine responses in IL-22^−/−^ mice after low dose *Mtb* infection. C57BL/6 mice (black bars) and IL-22^−/−^ (white bars) mice were infected with approx. 100 CFU *Mtb* via the aerosol route. At (**A**) earlier and (**B**) later time points, gene expression of *Il12b*, *Tnf*, *Il6*, and *Il10* was quantified by real time RT-PCR in lung homogenates of C57BL/6 mice based on the expression of *Hprt*. Data represent mean ± SD of 5 mice per group. Statistical analysis was performed using a two-way ANOVA defining differences between C57BL/6 and IL-22^−/−^ mice as significant (*, p<0.05).

IL-22 has been shown to mediate immunopathology in inflammatory diseases and to promote innate immune responses during intra- and extracellular bacterial or fungal infections [32], [34], [35], [54]–[57]. To evaluate the impact of IL-22 on the inflammatory immune response, the relative mRNA expression of pro- and anti-inflammatory cytokines (*Il12b*, *Tnf*, *Il6*, *Il10*) was quantified in lung homogenates of C57BL/6 and IL-22^−/−^ mice at earlier (Fig. 3A) and later (Fig. 3B) time points of low dose *Mtb* infection. Whereas expression of the IL-22-dependent genes *Mmp9* and *Cxcl10* [56], [57] was slightly reduced in IL-22^−/−^ mice (data not shown), gene expression of *Il12b*, *Tnf* and *Il10* was comparable in C57BL/6 and IL-22^−/−^ mice. Only the expression of *Il6* was slightly enhanced in IL-22^−/−^ mice at day 42. These results indicate that, after low dose infection with *Mtb* IL-22 is not required for the efficient induction of an inflammatory immune response.

### The Recruitment of Macrophages, Granulocytes, and Activation of T cells in IL-22^−/−^ Mice after Low Dose *Mtb* Infection

**Figure 4:**
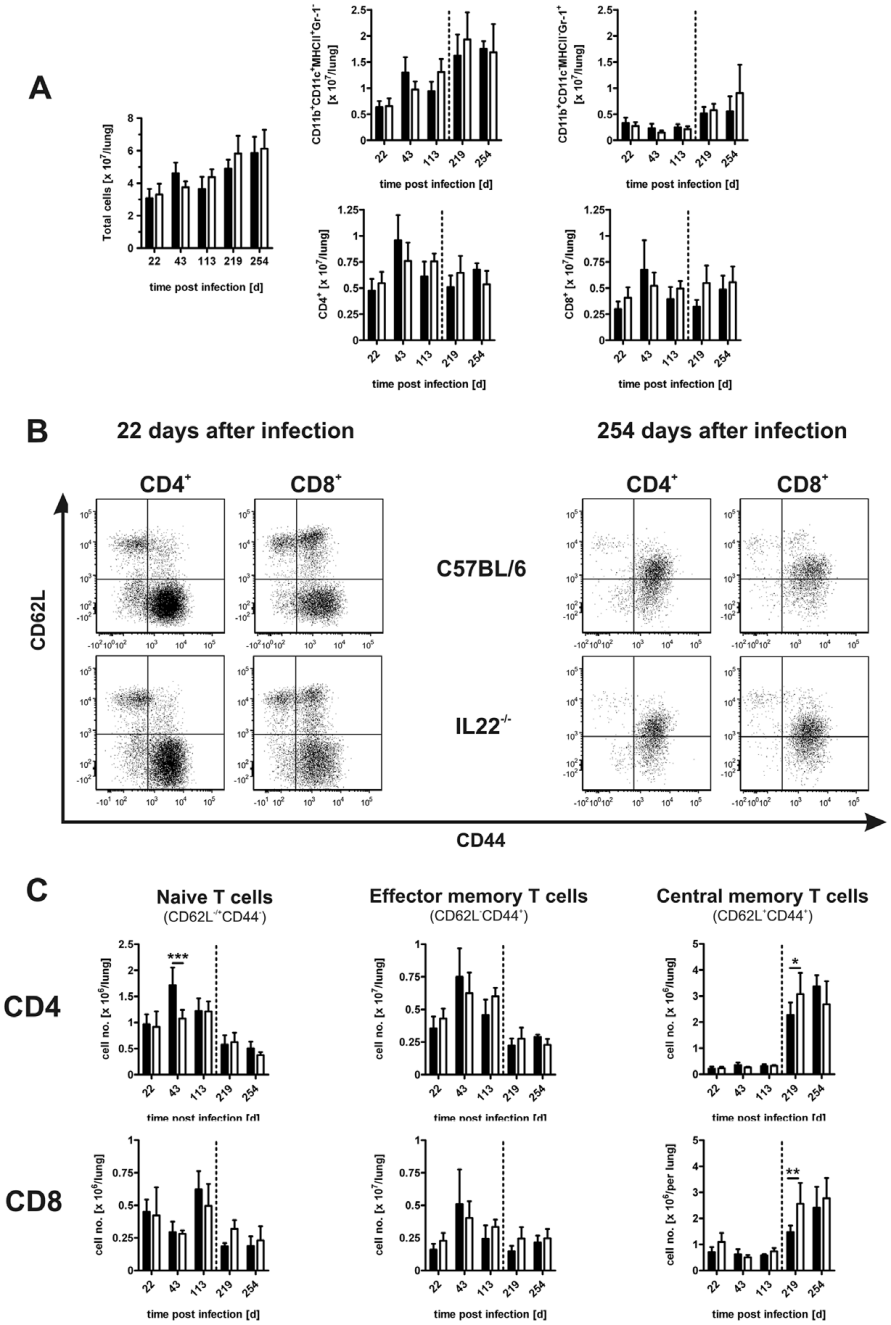
The recruitment of macrophages, granulocytes, and activation of T cells in IL-22^−/−^ mice after low dose *Mtb* infection. C57BL/6 mice (black bars) and IL-22^−/−^ (white bars) mice were infected with approx. 100 CFU *Mtb* via the aerosol route. At different time points, single cell suspensions of lungs were prepared, counted and stained for flow cytometric analysis. (**A**) Numbers of total infiltrating cells, macrophages (CD11b^+^CD11c^+^MHCII^+^Gr-1^neg^), granulocytes (CD11b^+^CD11c^neg^MHCII^neg^Gr-1^+^) and T cells (CD4^+^ or CD8^+^). (**B**) Representative dot plots of naive (CD4/8^+^CD62L^neg/+^CD44^neg^), effector memory (CD4/8^+^CD62L^neg^CD44^+^), and central memory (CD4/8^+^CD62L^+^CD44^+^) T cells at two representative time points (22 and 254 days after infection). (**C**) Analysis of T cell populations described in (**B**) during the course of *Mtb* infection. Data in (**A**) and (**C**) represent mean ± SD of 4–5 mice per group. Statistical analysis was performed using a two-way ANOVA defining differences between C57BL/6 and IL-22^−/−^ mice as significant (*, p<0.05; **, p<0.01; ***, p<0.001).

Effective recruitment of different leukocyte populations such as macrophages and lymphocytes in the lungs of infected mice is essential for the generation of a protective immune defence against *Mtb*. Therefore, we assessed the infiltration of these different cell populations into the lungs of C57BL/6 and IL-22^−/−^ mice at early and late time points of low dose *Mtb* infection (Figure 4). The total number of cells recruited to the lungs of IL-22^−/−^ mice was not significantly different to those found in the lungs of C57BL/6 mice (Figure 4A). During the course of *Mtb* infection, the amounts of macrophages (CD11b^+^CD11c^+^MHCII^+^Gr-1^neg^), granulocytes (CD11b^+^CD11c^neg^MHCII^neg^Gr-1^+^), and T cells (CD4^+^ and CD8^+^) were also comparable in both mouse strains (Figure 4A). Central and effector memory T cells play an important role to sustain adaptive immune responses during long term *Mtb* infection [58], [59]. To examine the development of memory T cells we defined three different CD4^+^ and CD8^+^ T cell subsets by flow cytometry (Figure 4B): naive T cells (CD62L^neg/+^CD44^neg^), effector memory T cells (CD62L^neg^CD44^+^), and central memory T cells (CD62L^+^CD44^+^). For both, C57BL/6 and IL-22^−/−^ mice, we saw similar results (Figure 4C) during the course of *Mtb* infection at days 22, 43, 113, 219, and 254. Compared to C57BL/6 mice, the numbers of naive as well as of effector memory T cells were slightly decreased in the lungs of IL-22^−/−^ mice, whereas the amounts of central memory T cells increased at later time points. However, significant differences between C57BL/6 and IL-22^−/−^ mice were only seen at d43 and d219 of infection with reduced numbers of naive CD4^+^ T cells and increased amounts of central memory T cells in mutant mice, respectively.

### The Development of TH1 and TH17 Immune Responses in *Mtb*-infected IL-22^−/−^ Mice

**Figure 5:**
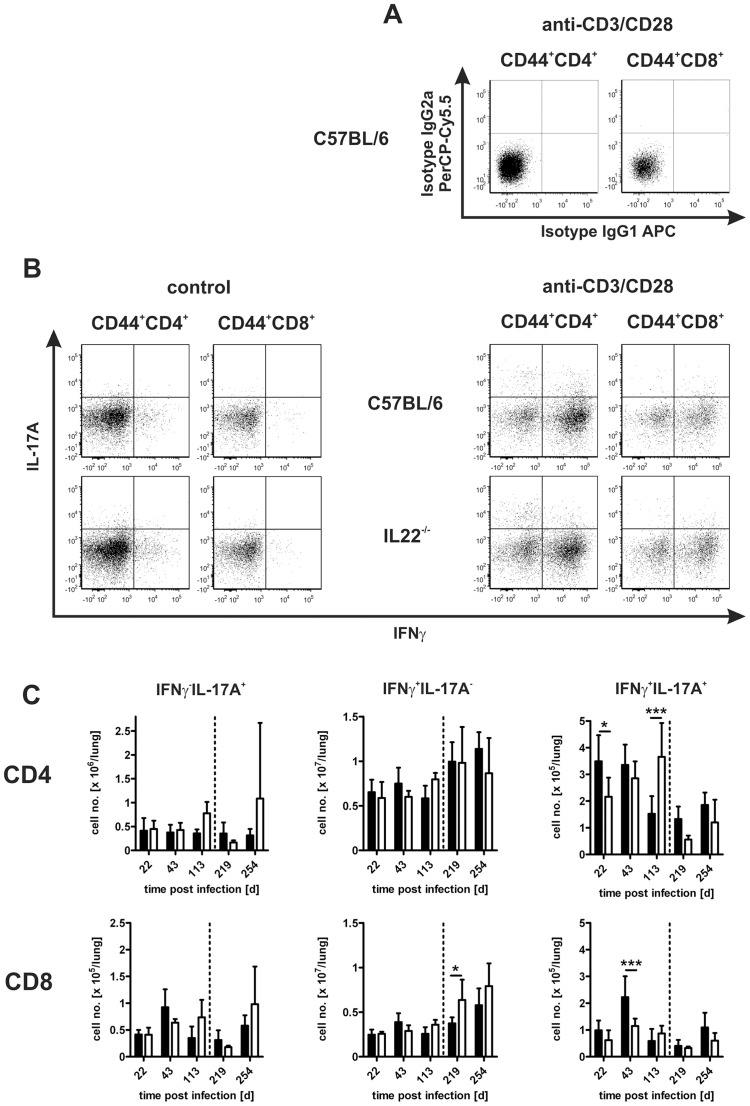
TH1 and TH17 immune responses in IL-22^−/−^ mice after low dose infection with *Mtb*. C57BL/6 mice (black bars) and IL-22^−/−^ (white bars) mice were infected with approx. 100 CFU *Mtb* via the aerosol route. At different time points, single cell suspensions of lungs were prepared, restimulated with anti-CD3/CD28 and stained for flow cytometric analysis. (**A**) Representative dot plots of restimulated cells from C57BL/6 mice stained with isotype control antibodies. (**B**) Representative dot plots of unstimulated (left panel) or restimulated (right panel) IFNγ- and IL-17A-producing CD44^+^CD4^+^ and CD8^+^ cells isolated at day 22 after infection. (**C**) Frequencies of IFNγ^neg^IL-17A^+^, IFNγ^+^IL-17A^neg^, and IFNγ^+^IL-17A^+^CD4/CD8^+^ cells. Data represent mean ± SD of 4–5 mice per group. Statistical analysis was performed using a two-way ANOVA defining differences between C57BL/6 and IL-22^−/−^ mice as significant (*, p<0.05; ***, p<0.001).

A TH1 immune response is central to macrophage activation and the subsequent elimination of intracellular mycobacteria by the production of IFNγ. However, IL-17A-secreting TH17 cells have also implicated in promoting protective effector mechanisms [25], [28]. To determine the capability of CD4^+^ and CD8^+^ cells to produce IFNγ or IL-17A in the absence of IL-22, we performed intracellular cytokine staining and flow cytometric analysis of lymphocytes isolated from lungs of C57BL/6 and IL-22^−/−^ mice at different time points of low dose *Mtb* infection. Lung cells were incubated with medium alone or were restimulated with plate bound anti-CD3/CD28. Unstimulated suspensions did not contain appreciable frequencies of IFNγ- or IL-17A-producing CD4^+^ or CD8^+^ cells, but upon stimulation three cytokine-producing CD4^+^ and CD8^+^ populations were detectable (Fig. 5A): IFNγ^neg^IL-17A^+^, IFNγ^+^IL-17A^neg^, and IFNγ^+^IL-17A^+^. In general, no differences between C57BL/6 and IL-22^−/−^ mice in the frequencies of these cell populations were detected (Figure 5B). At some time points slightly different frequencies of CD8^+^IFNγ^+^IL-17A^neg^, CD4^+^IFNγ^+^IL-17A^+^, CD8^+^IFNγ^+^IL-17A^+^, and CD4^+^IFNγ^+^IL-17A^+^ cells were found (Figure 5B). However, these differences between C57BL/6 and IL-22^−/−^ mice were not consistent during the course of low dose *Mtb* infection.

**Figure 6:**
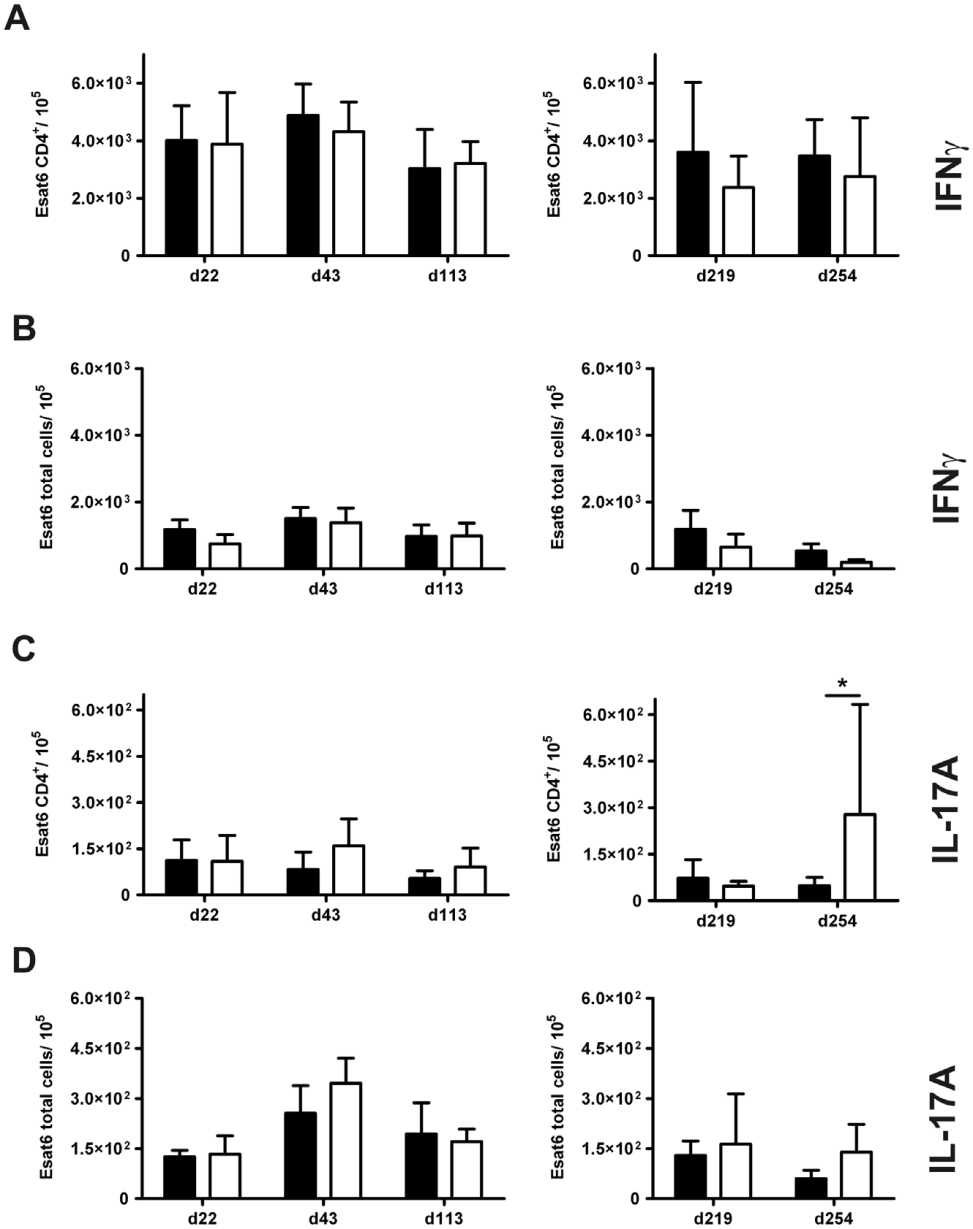
Antigen-specific production of IFNγ and IL-17A by CD4^+^ T cells of low dose-infected IL-22^−/−^ mice. C57BL/6 mice (black bars) and IL-22^−/−^ (white bars) mice were infected with approx. 100 CFU *Mtb* via the aerosol route. At earlier (left panel) and later (right panel) time points, the frequency of Esat-6_1–20_ specific (**A**, **B**) IFNγ- and (**C**, **D**) IL-17A-producing cells in (**A**, **C**) cell suspensions enriched for CD4^+^ T cells or in (**B**, **D**) whole cell suspensions from lungs was determined by ELISPOT assay. Data represent mean ± SD of 4–5 mice per group. Statistical analysis was performed using a two-way ANOVA defining differences between C57BL/6 and IL-22^−/−^ mice as significant (*, p<0.05).

We next evaluated antigen-specific TH1 and TH17 immune responses in C57BL/6 and IL-22^−/−^ mice at early (Figure 6A–D, left panel) and late (Figure 6A–D, right panel) time points of low dose *Mtb* infection. The frequency of ESAT6_1–20_-specific IFNγ- and IL-17A-producing TH1 and TH17 cells, respectively, were determined within an enriched CD4^+^ cell population (Figure 6A, C) and within total cell suspensions (Figure 6B, D) isolated from lungs of infected mice by ELISPOT assays. During the course of low dose infection, both, C57BL/6 and IL-22^−/−^ mice generated similar frequencies of *Mtb*-specific TH1 and TH17 cells within the CD4^+^ cell population and within total lung cell suspensions. However, at day 254 of infection, *Mtb*-specific IL-17A-producing CD4^+^ T cells were significantly enhanced in IL-22^−/−^ mice.

Together, these results reveal that IL-22 has no influence on the expression of TH1 and TH17 immune responses of a low dose *Mtb* infection.

### Macrophage Activation in *Mtb*-infected IL-22^−/−^ Mice

**Figure 7:**
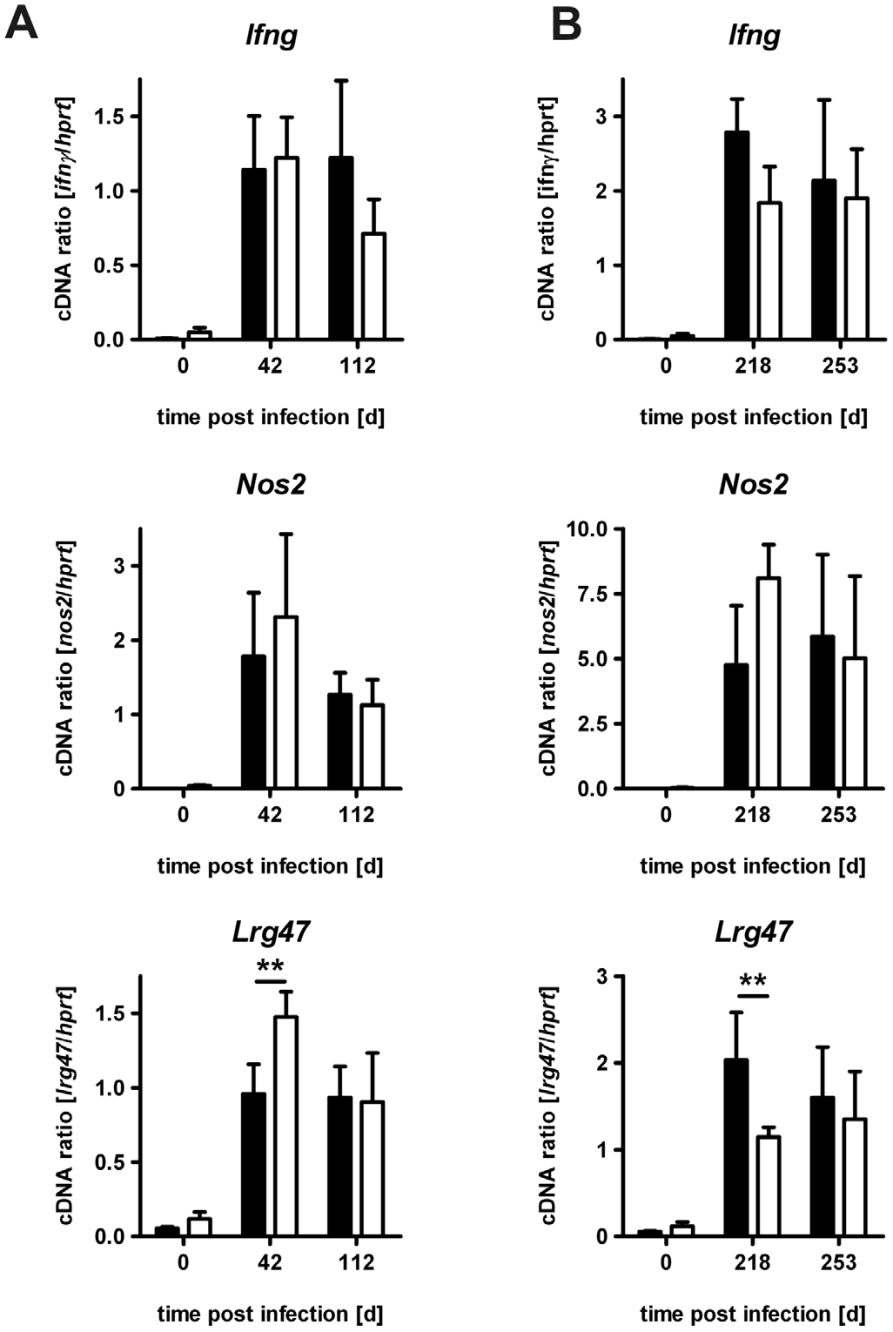
Macrophage effector response in IL-22^−/−^ mice after low dose *Mtb* infection. C57BL/6 mice (black bars) and IL-22^−/−^ (white bars) mice were infected with approx. 100 CFU *Mtb* via the aerosol route. At (**A**) earlier and (**B**) later time points, gene expression of *Ifng*, *Nos2*, and *Lrg47* was quantified by real time RT-PCR in lung homogenates of C57BL/6 mice based on the expression of *Hprt*. Data represent mean ± SD of 5 mice per group. Statistical analysis was performed using a two-way ANOVA defining differences between C57BL/6 and IL-22^−/−^ mice as significant (**, p<0.01).

So far we have shown that after low dose infection with *Mtb* IL-22 is not essential for the efficient generation of inflammatory immune responses and the subsequent induction of antigen-specific TH1 and TH17 cells. However, IL-22^+^CD4^+^ T cells are capable to inhibit intracellular *Mtb* replication in monocyte-derived macrophages from macaques [60]. In mice, the IFNγ-dependent expression of NOS2 and LRG-47 are absolutely required to control *Mtb* growth in macrophages [61], [62]. Therefore, we examined the induction of these pathways in lung homogenates of C57BL/6 and IL-22^−/−^ mice after low dose *Mtb* infection by real time RT-PCR. Gene expression of *Ifng*, *Nos2* and *Lrg47* were considerably induced after infection, but no differences between the expression of these genes in C57BL/6 and IL-22^−/−^ were found during the course of low dose *Mtb* infection (Figure 7). However, gene expression of *Lrg47* was slightly modulated at days 42 and 218 after *Mtb* infection. Overall these results indicate a less important role of IL-22 for promoting effector functions in macrophages.

### The Absence of IL-22 does not Affect the Outcome of *Mtb* Infection

**Figure 8:**
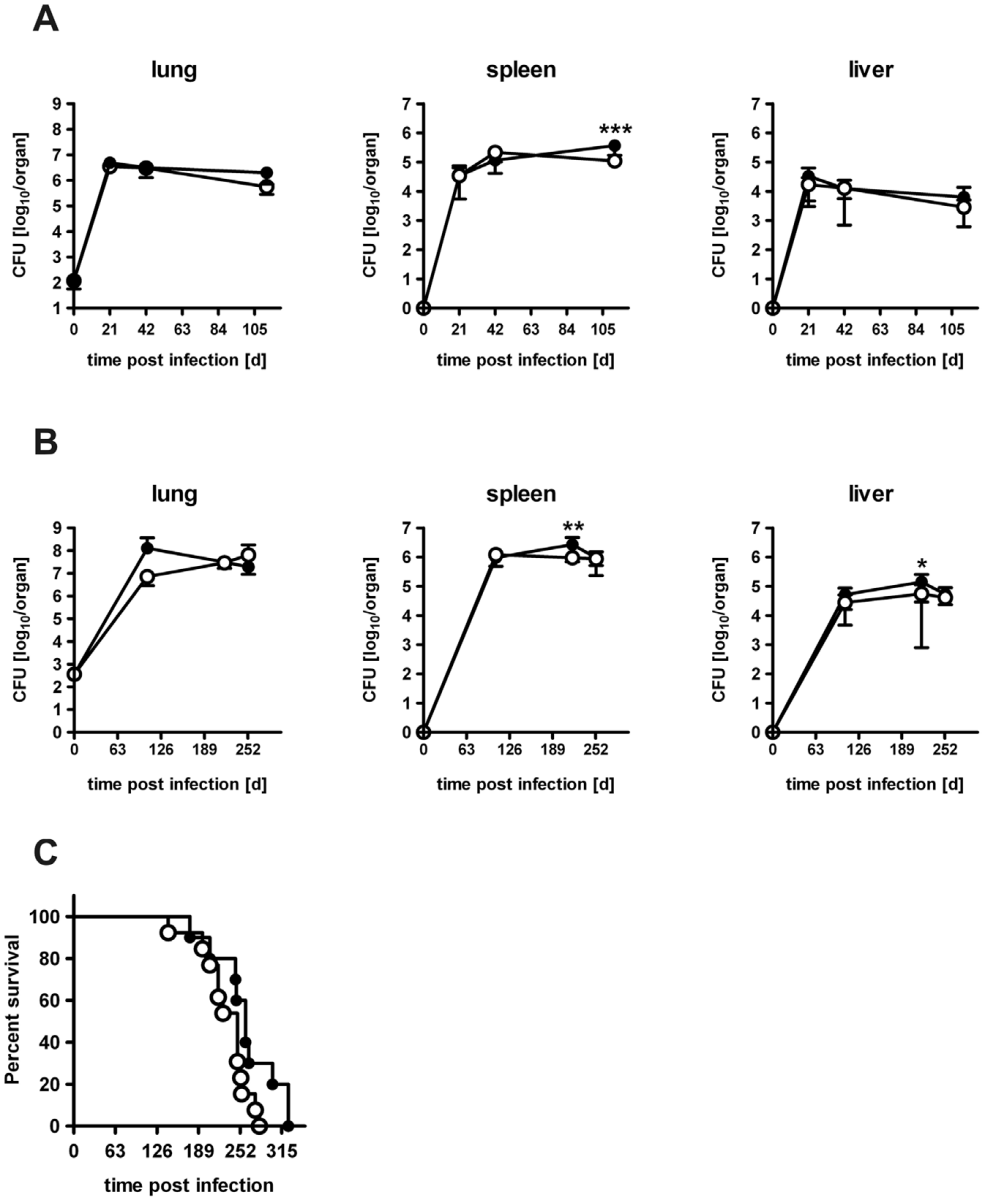
The course of low dose *Mtb* infection in IL-22^−/−^ mice. C57BL/6 mice (black symbols) and IL-22^−/−^ (white symbols) mice were infected with approx. 100 CFU *Mtb* via the aerosol route. At (**A**) earlier and (**B**) later time points, mycobacterial colony enumeration assays were performed in lungs, spleen, and liver. Data represent mean ± SD of 4–5 mice per group. Statistical analysis was performed using two-way ANOVA defining differences between C57BL/6 and IL-22^−/−^ mice as significant (*, p<0.05; **, p<0.01; ***, p<0.001). (**C**) Survival of 10–13 infected mice per group. Statistical analysis was performed using the log-rank test.

**Figure 9:**
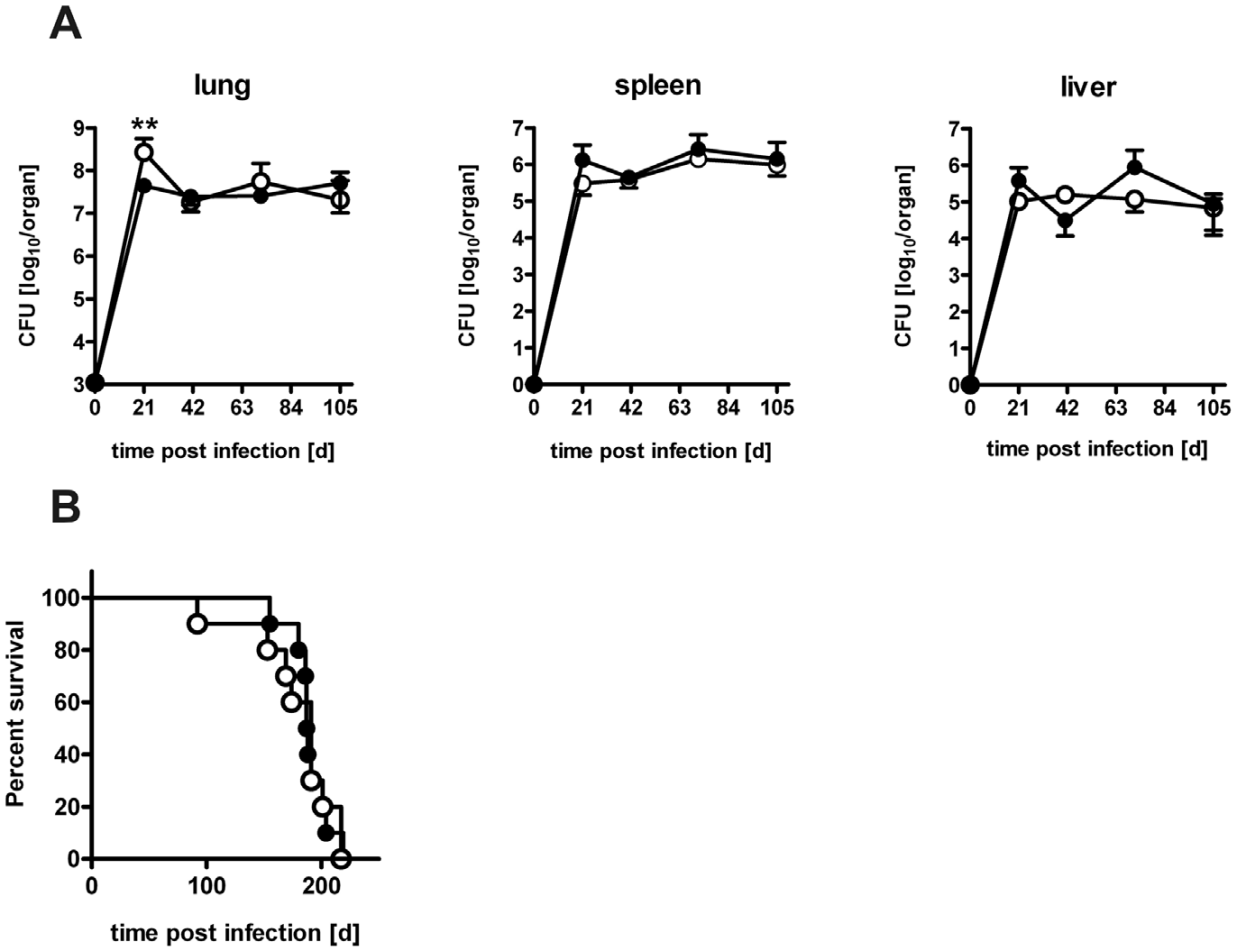
The course of high dose *Mtb* infection in IL-22^−/−^ mice. C57BL/6 mice (black symbols) and IL-22^−/−^ (white symbols) mice were infected with approx. 1000 CFU *Mtb* via the aerosol route. At (**A**) earlier and (**B**) later time points, mycobacterial colony enumeration assays were performed in lungs, spleen, and liver. Data represent mean ± SD of 5 mice per group. Statistical analysis was performed using two-way ANOVA defining differences between C57BL/6 and IL-22^−/−^ mice as significant (**, p<0.01). (**C**) Survival of 10 infected mice per group. Statistical analysis was performed using the log-rank test.

To finally evaluate whether IL-22 has any influence on the outcome of experimental TB, C57BL/6 and IL-22^−/−^ mice were infected with low doses of *Mtb* via the aerosol route and the course of infection was followed (Figure 8). However, bacterial loads in the lungs of IL-22^−/−^ mice did not significantly differ from lungs of C57BL/6 mice at early (Figure 8A) and late (Figure 8B) time points of infection. In spleens and livers of IL-22^−/−^ mice, even slightly reduced CFU were found at some time points of *Mtb* infection. In line with this apparently efficient control of *Mtb* replication in the absence of IL-22 also no difference in survival kinetics was observed between IL-22^−/−^ and C57BL/6 mice (Fig. 8C). These results clearly show that IL-22 is dispensable for controlling low dose *Mtb* infection. To evaluate whether IL-22 is required for protective immune responses after high dose *Mtb* infection, we infected mice with more than 1000 CFU *Mtb* and followed the course of infection (Figure 9A). On day 41, 71, and day 105 after infection the bacterial load in the lungs of L-22^−/−^ mice did not significantly differ from lungs of C57BL/6 mice, showing that IL-22^−/−^ mice controlled bacteria as well as C57BL/6 mice up to 105 days. Earlier during infection (d21), only lungs of IL-22^−/−^ mice could worse with *Mtb* infection than C57BL/6 mice showing slightly higher (∼0.6 log_10_) bacterial loads. No differences were found in the spleens and livers of *Mtb* infected C57BL/6 and IL-22^−/−^ mice. Additionally performed flow cytometric analysis revealed no difference within the amount of T cells, macrophages, and granulocytes during the course of infection (data not shown). Furthermore, the cell-mediated immune response measured by real time RT-PCR and ELISPOT analysis was not significantly different in high dose *Mtb*-infected C57BL/6 and IL-22^−/−^ mice (data not shown). In line with these findings, also no difference in survival kinetics was observed between IL-22^−/−^ and C57BL/6 mice after high dose *Mtb* infection (Figure 9B). Together, these results indicate that IL-22 is dispensable for protective immune responses during TB.

## Discussion

IL-22, a member of the IL-10 family, is mainly produced by T and NK cells [33], [38]. It is considered to be produced by IL-17A-secreting TH17 cells in an IL-23-dependent manner [20], [32] or by a private T cell lineage termed TH22 [63], [64]. In patients with inflammatory skin disorders, these TH22 cells were found to produce only IL-22 but not IFNγ, IL-4, or IL-17 [63]. We here show that after infection of C57BL/6 mice with *Mtb*, IL-22 expression is in fact dependent on IL-23. Most of the IL-22-expressing cells also produce IFNγ (>90%). To a minor extent T cells secret only IL-22 but co-expression by IL-17A-producing cells was hardly detectable. Hence, in contrast to current paradigm of T cell lineages defining IL-22 produced by TH17 or TH22 cells [64] IL-22 appears to be a TH1 cytokine during experimental TB. Based on *in vitro* studies TH17 cells have been thought for a long time to represent terminally differentiated cells but recent reports facilitated by novel tools for tracking TH17 cell fate *in vivo* give clear evidence of a developmental plasticity of TH17 cells [65], [66]. Because the discovery of IL-17A/IFNγ co-producing cells has originally indicated a plasticity between TH17 and TH1 differentiation [17] and a recent study that analyzed TH17 cell fate during chronic inflammation *in vivo* demonstrated an IL-23-mediated conversion of TH17 to TH1 cells [67], IL-23-dependent IL-22-expressing TH1 cells described in the present study might represent a transition state of TH22 to TH1 cells. In *Mtb* infected C57BL/6 mice, most of the IFNγ^+^IL-22^+^ T cells were of the CD4 and CD8 T population but IL-22-single positive cells were mainly produced by CD4 T cells and could be considered to represent TH22 cells. A fairly prominent proportion of IL-22-single positive cells (approx. 30%) were found to be CD4^neg^CD8^neg^CD90.2^+^ and may belong to the NK cell population, that has recently be described as IL-22 producing cells after *Mtb* infection [47]. Together, during experimental TB IL-22 is mainly produced by TH1 and TH22 cells rather than by TH17 cells and that IL-22 expression depends on IL-23. The fate of IL-22-producing cells and the potential transition into TH1 cells has to be further elucidated. *Mtb* infection usually results in a TH1-dominated immune response. Because TH1 cells are potent regulators of the development of IL-17A/IL-22 co-producing TH17 cells [68], the induction of TH1 cytokines may also suppress the efficient generation of TH17 cells expressing both IL-17A and IL-22 and favours the initiation of IFNγ/IL-22 co-producing cells. This bypass allows for an effective IL-22 expression without co-secretion of IL-17A.

IL-22 signals through a receptor complex, consisting of the specific IL-22 receptor type I and the IL-10R2 subunit and activates the STAT3 signaling pathways, and is capable to induce pro-inflammatory chemokines or cytokines, antimicrobial peptides, and proteins involved in tissue remodeling [32], [33], [37], [38], [64]. However, in the present study gene expression of *il12b*, *tnf*, *il6*, and *il10* in lung of IL-22^−/−^ mice was efficiently induced after aerosol infection with *Mtb*. Hence, the inflammatory cytokine response during TB is generated independently of IL-22. The effective recruitment of different leukocyte populations in the lungs of infected mice is essential for the generation of a protective immune response against *Mtb* [69]. We here show that during the course of *Mtb* infection the infiltration of macrophages, granulocytes and T cells into the lungs is facilitated independently of IL-22. Memory T cells sustain adaptive immune responses during the course of experimental TB [58]. In the present study, the differential analysis of naive, effector memory and central memory T cells also revealed that IL-22 has no general influence on the development of memory and central memory T cells after infection with *Mtb*. Reduced numbers of naive CD4^+^ T cells at early stages of infection day may be explained by slightly reduced expression of the IL-22-dependent chemokines MMP-1 and CXCL-10 in IL-22^−/−^ mice, which are involved in activation and recruitment of T cells [56], [57], [70], [71] (data not shown). In line with the rather unaffected development of memory and central memory T cells in *Mtb*-infected IL-22^−/−^ mice the differentiation of IFNγ^+^IL-17A^+^ T cells as well as of TH1 and TH17 cells was also fairly induced in the absence of IL-22. Irrespective of the IL-22-independent induction of inflammatory cytokines, leukocyte recruitment and T cell activation, IL-22 might activate macrophage effector mechanisms, because it has been described in different models to limit mycobacterial growth in these cells [48], [60]. However, in the present study the expression of important macrophage effector molecules such as NOS2 and LRG-47 [61], [62] was efficiently induced independently of IL-22. Together, our detailed analysis revealed that during experimental TB IL-22 is not essential for the generation of a cell-mediated immune response. Because IL-22 expression depends on the presence of IL-23 this phenotype very much corroborates the still efficient immune response against *Mtb* infection described in IL-23p19^−/−^ mice [20].

The antimicrobial activity against several other intra- and extracellular pathogens *in vivo* is promoted by IL-22 [47], [55], [57] and also the regenerative and inflammatory effect of this cytokine is documented during influenza infection [72]. With respect to TB, IL-22 produced by CD4 T or NK cells is capable to inhibit the growth of *Mtb* in macrophages *in vitro* [48], [60] indicating a protective function *in vivo*. However, in another context in which different infectious diseases and other cytokines were specifically evaluated IL-22^−/−^ mice were resistant to mycobacterial infection [48], [49], [53]. Focusing on IL-22, our detailed analysis revealed that after infection with *Mtb* IL-22 has no influence on the expression of inflammatory cytokines, cellular infiltration, the expansion of memory T cells, the development of TH1 and TH17, and the expression of macrophage effector functions. Consequently, IL-22^−/−^ mice were capable to efficiently control mycobacterial growth after low dose infection with a similar survival kinetic than infected C57BL/6 mice. Even after high dose challenge with *Mtb* an IL-22 deficiency has no impact on the outcome of experimental TB. Together, IL-22 alone is dispensable for host protective immune responses against *Mtb* and in the complex *in vivo* system of the here presented study IL-22 deficiency appears to be compensated by other mediators. This corroborates again the phenotype of IL-23p19^−/−^ mice in which IL-23 deficiency is compensated by IL-12 [20]. Hence, other IL-23-dependent mediators such as IL-17A or IL-17F may therefore replace functions of IL-22. Antimicrobial peptides, associated with host defense, are cooperatively induced by IL-22, IL-17A and IL-17F, but IL-22 alone is not as strong as IL-17A or IL-17F in inducing these peptides [32]. This potential compensatory effect may eventually aid to the efficient control of mycobacterial growth in *Mtb*-infected IL-22^−/−^ mice.

Our findings that IL-22 is not essential for protective immune responses against *Mtb* provide evidence that IL-22-targeted therapeutics in inflammatory diseases may not incur the risk of adverse effects such as reactivation of TB.
